# Microscopic anatomy of the carotid canal and its relations with cochlea and middle ear

**DOI:** 10.1016/S1808-8694(15)31191-5

**Published:** 2015-10-20

**Authors:** Norma de Oliveira Penido, Andrei Borin, Yotaka Fukuda, Cristina Navarro Santos Lion

**Affiliations:** 1 Ph.D. in Medicine, Affiliated Professor, UNIFESP-EPM.; 2 Master in Otorhinolaryngology, UNIFESP/EPM, Otorhinolaryngologist.; 3 Full Professor, Associate Professor, Department of Otorhinolaryngology, UNIFESP/EPM.; 4 Master in Otorhinolaryngology, UNIFESP/EPM, Otorhinolaryngologist. Department of Otorhinolaryngology and Head and Neck Surgery, Federal University of Sao Paulo - Escola Paulista de Medicina (UNIFESP/EPM) and House Ear Institute (Los Angeles, CA).

**Keywords:** anatomy, temporal bone, internal carotid artery, cochlea, ear

## Abstract

The knowledge of the relations between the noble and vital structures of temporal bone is still a great challenge for the otologic surgeon. The microscopic anatomic studies of the temporal bone are one of the greatest help to prevent lesions during surgical intervention. **Aim**: To study the anatomic correlations between the carotid canal and the cochlea, and the occurrence of dehiscence of the carotid canal in the middle ear tympanic cavity. **Material and Methods**: Microscopic study of 122 human temporal bones. RESULTS: The average distance between the carotid canal and the cochlea were: the shortest distance, 1.05mm; basal turn, 2.04mm; middle turn, 2.32mm; and apical turn, 5.70mm. The occurrence of dehiscence of the carotid canal inside the tympanic cavity was 35.2%. **Conclusion**: The small distances between the cochlea and carotid canal, and the high incidence of dehiscence in the tympanic cavity remind us that anatomical knowledge of the temporal bone is required for the best qualification of otologists.

## INTRODUCTION

Anatomic knowledge has always been something indispensable in medical activity. The knowledge of anatomical structures present in the temporal bone and its relations take on a key role owing to their complexity, in which many vital and important structures are extremely close one to the other. This three-dimensional anatomical notion is essential to train the Otorhinolaryngologist, especially those that work in the surgical area, involved with the treatment of intrinsic or invasive diseases of the temporal bone, as well as using it as access route to the central nervous system.

Embryologically, the circulation system of the carotid artery has its origin in the branchial vessels, and the 3rd arch artery is originated from the internal carotid artery ^1^. The internal carotid artery may be divided into 4 parts: cervical, petrous, cavernous and cerebral. The petrous portion has its pathway involved in one single bone canal inside the temporal bone and it has two segments. One vertical segment - ranging from 5.0 to 12.5mm long and 4.0 to 7.5mm in diameter, defining a correlation, posteriorly, with the jugular fossa, anteriorly, with the auditory tube, and anterior-laterally with the tympanic bone. In the anterior-inferior region, the chlocleoform process, extremely close to the cochlear turns, has a change in direction, starting its horizontal segment that is directed anterior-medially by a path of 14.5 to 24mm long and 4.5 to 7.0mm in diameter.

Microanatomic studies allow the surgeons to have full anatomic orientation over the temporal bone region in which they are working, which comprises many anatomical vital structures, whose unexpected damage would be catastrophic. Among them, internal carotid artery (ICA) lesion represents a transoperative bleeding risk that is difficult to control, in addition to leading to severe consequences in relation to the irrigation of the central nervous system. In view of that, we present a microscopic anatomic study of the relations between the carotid canal (CC) and cochlear turns and also the analysis of the occurrence of dehiscence close to the tympanic cavity (TC).

## METHOD

The base of this study was human temporal bones that belonged to the Temporal Bone Bank of Laboratory George Eccles, located at House Ear Institute, in Los Angeles (California, USA). All of the bones had been donated in life by the patients, comprising a complete medical history about otological diseases. It allowed the definition of strict inclusion and exclusion criteria for the selection of analyzed temporal bones. We excluded all bones that could have had anatomical affections resultant from diseases that theoretically could imply distortions of microscopic anatomy, such as congenital malformations, otosclerosis, Paget, temporal fractures, etc. The temporal bones, removed some hours after the death of the patients, were prepared in the horizontal plan with 20-micrometer sections, comprising the ampulla of the superior semicircular canal to the end of the posterior semicircular canal. They were later stained with hematoxyllin and eosin to be analyzed under optical microscope. The technique for harvesting, preparation, section and staining is described in the manual of the institution ^2^.

**Figure 1:**
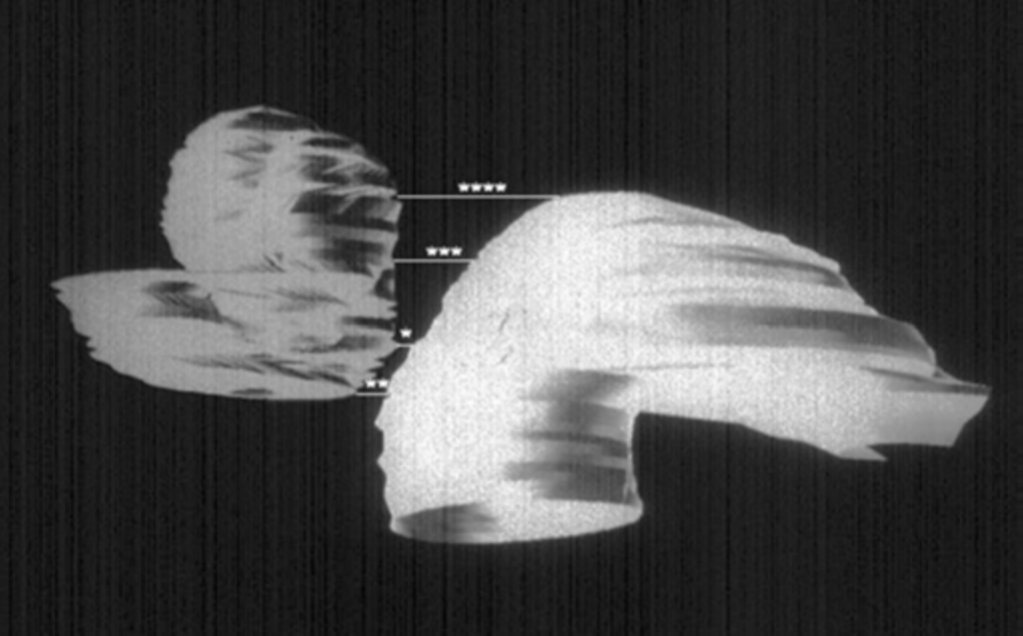
Three-dimensional reconstruction based on histologic sections of the same temporal bone, made by the computer, of the cochlea and the carotid canal, representing the sites where the measurements were made: *shortest distance, ** basal turn, *** medium turn, **** apical turn.

**Figure 2:**
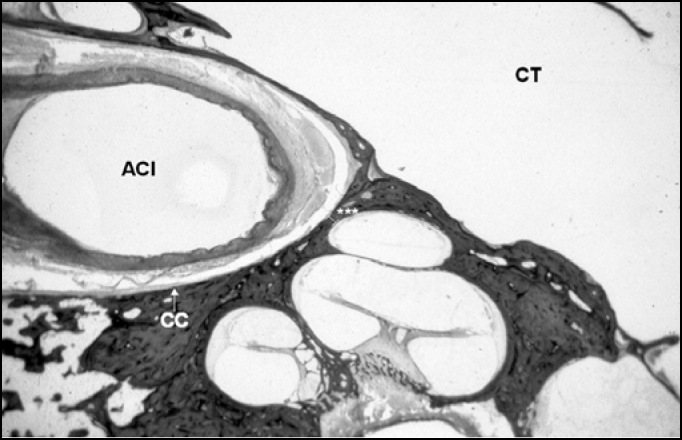
Smallest amount of the measurement made between the carotid canal and the apical turn (0.19 millimeters). CC = Carotid canal; ACI = Internal Carotid Artery; CT = Tympanic Cavity. Histological section enlarged 8X.

**Figure 3:**
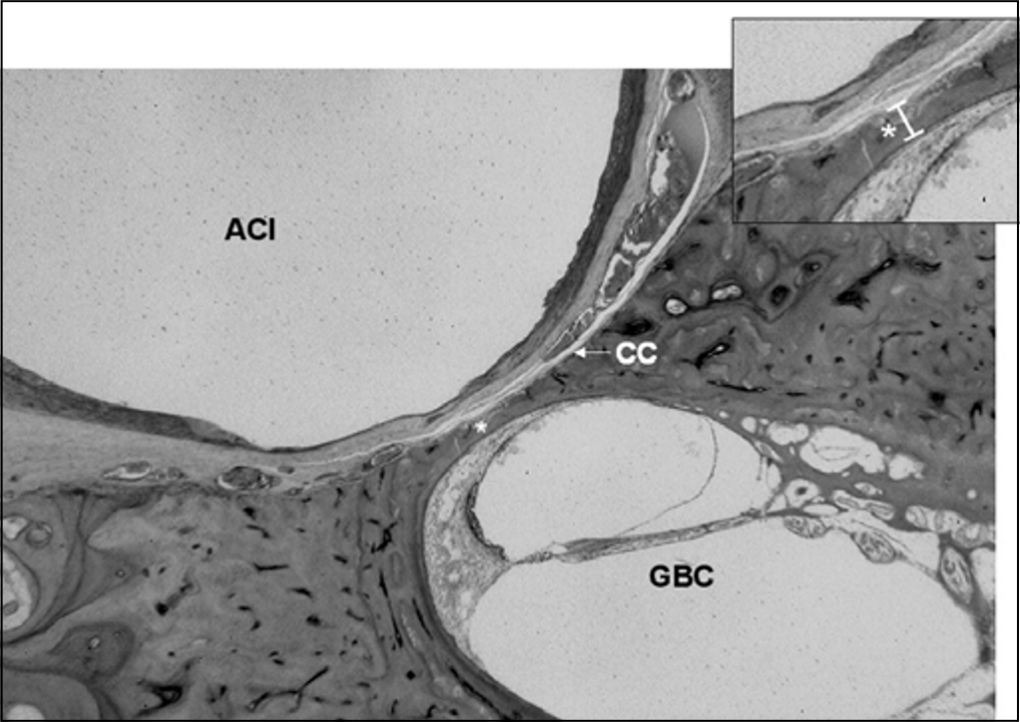
Smallest value of the measurement between the carotid canal and the basal turn (0.12 millimeters). CC = Carotid canal; ACI = Internal Carotid Artery, GBC = Cochlear Basal Turn. Histological section enlarged 20X.

Two analyses of the microanatomy of the carotid canal in its intratemporal pathway were performed. The first analysis involved measurement of the minimum distance, in millimeters, from the carotid canal to the cochlea. To that end, we performed measurements in four cochlear regions (Figure 1), which were: first measurement to determine the site of the smallest distance between the carotid canal and the cochlear optic capsule (Figures 2 and 3), a measurement that could have been located in any region of the cochlea (basal turn, medium turn or apical turn); second measurement at the basal turn (located in the basal turn specifically in the last slide in which there was the cochlea and the carotid canal); this was the last slide because the sections were made from top down in the temporal bone; third measurement in the medium turn (corresponds to the site of the medium turn between the carotid canal and the otic capsule of the cochlea on the slide with presence of modiolus axis), and the fourth measurement of the apical turn (corresponding to the region of the cochlea apex on the first slide in which the section goes through the cochlea, a measurement which was made only when the carotid canal passed through this level). Two independent researchers made measurements that were statistically analyzed. The following statistical tests were analyzed: Wilcoxon Test, Mann-Whitney Test, Spearman correlation coefficient. Taking into account the size of the sample, the tests were applied based on the normal curve.

**Figure 4:**
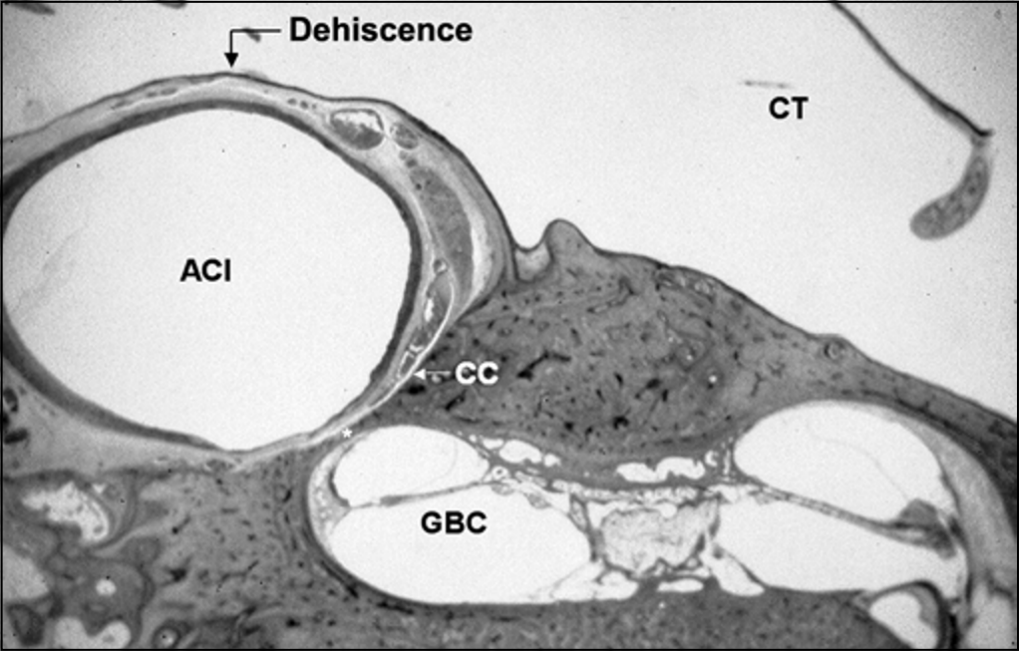
Histologic section of temporal bone showing the large dehiscence of the carotid canal in the tympanic membrane. Total absence of carotid-tympanic wall between the internal carotid artery (ACI) and the tympanic cavity (CT).

**Figure 5:**
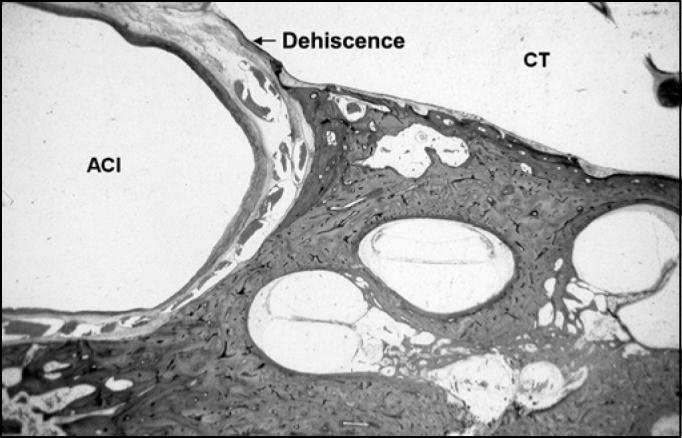
Histologic section of the temporal bone showing a small dehiscence of carotid canal in the tympanic cavity. Absence of small portion of the carotid tympanic wall between the internal carotid artery (ACI) and the tympanic cavity (CT).

The second analysis involved the assessment of the presence of bone dehiscence of the CC in the TC, defined as absence of bone coverage of the intima in the carotid artery in some histological sections (Figures 4 and 5). These data were later correlated with information concerning patients’ gender and bone laterality.

## RESULTS

Out of a total of 600 temporal bones available in the bank of bones, we used to this study, according to the criteria described in the methodology, 122 temporal bones, being that 51 were paired and 22 were non-paired. There were 30 female paired bones and 13 female non-paired bones, and 21 paired and 7 non-paired male bones. The age at death of the donors ranged from 6 to 97 years.

Our results concerning the distance between cochlear turns and CC are shown in Table 1, separated by laterality and gender. Given that there was no statistically significant difference (p<0.05) between the measurements made by the two researchers, concerning gender and laterality, we decided to analyze them together. Thus, we could see that on average CC was 1.05mm away from the cochlea at its minimum distance; in the basal cochlear turn, 2.04mm, in the medium turn, 2.32mm, and in the apical turn, 5.77mm. We should emphasize that the measurements of the region of the apical turn could be made in only half of the cases owing to changes in direction of CC in this region.

**Table 1:** Mean distance of carotid canal and cochlear turns, expressed in millimeters, and minimum and maximum values found in the samples.

	Male Gender		Female Gender		GENERAL	
	Right Ear	Left Ear	Right Ear	Left ear	General Mean	
**Small value**		1,09	1,07	1,06	0,98	1,05
	(0,12-3,40)	(0,123,14)	(0,19-3,05)	(0,14-2,38)	(0,15-3,00)	
**Basal**	2,20	2,04	2,09	1,87	2,04	
	(0,76-4,33)	(0,33-4,33)	(0,38-4,04)	(0,52-3,76)	(0,33-4,33)	
**Medium**	2,28	2,49	2,38	2,24	2,32	
	(0,71-5,66)	(0,71-6,57)	(0,47-9,19)	(0,48-5,23)	(0,47-9,19)	
**Apical**	6,36	5,58	5,59	5,46	5,77	
	(4,38-7,95)	(4,95-6,66)	(4,14-7,95)	(2,24-7,95)	(2,24-7,95)	

As to occurrence of CC dehiscence in the TC, our results are described in Table 2. Once again, given that there was no statistically significant difference (p<0.05) concerning gender and laterality, we decided to analyze them together. We detected the occurrence of CC dehiscence in the TC in 35.2% of the temporal bones.

**Table 2:** Occurrence of carotid canal dehiscence in the middle ear (number of temporal bones in the sample is presented in brackets).

	Male (49)	Female (73)	TOTAL (122)
Right Ear (62)	14,3% (7)	19,2% (14)	33,9% (21)
Left Ear (60)	14,3% (7)	20,5% (15)	36,7% (22)
TOTAL (122)	28,6% (14)	39,7% (29)	35,2% (43)

## DISCUSSION

Many different otological surgeries involve temporal bone dissection close to the CC, adjacent to the cochlea. Among them, we can highlight apicectomy, transcochlear access to the cerebello-pontine angle, transotic access and temporalectomy 3, 4, 5. Our study calculated the mean distances between CC and cochlear turns: in the smallest site (1.05mm), in basal turn (2.04mm), in the medium turn (2.32mm) and in the apical turn (5.77mm). The literature confirms our findings: Paullus et al.^6^ defined the average distance between cochlear basal turn and CC as 2.1mm (ranging from 0.6 to 10mm); Muren et al.^7^ as 1.34mm (0.2 to 6.2mm); and Lang^8^ as 2.2mm (0.4 to 7.0). These small values between CC and cochlear turns warn us about the proximity of these structures, which should always be remembered during temporal bone surgical dissection. Especially in elderly people, in which ICA walls may suffer the process of muscle absorption, which results in special frailty, in which the CC may ensure the integrity of blood flow ^9^.

Our sample involved patients of a wide age range, a fact that apparently does not negatively influence our study, given that in the literature we can find the report that at birth the labyrinthic block is already fully developed, respecting the dimensions of the adult 7, 9, 10.

The origin of CC dehiscence in the TC may have many possible explanations such as failures in ossification, congenital anomaly, persistence of embryonic vessels, bone absorption throughout the years, or middle ear inflammatory processes 11, 12, 13, 14. They represent an additional risk during otological surgeries and they may lead to accidental damage of ICA. The microanatomical study showed incidence of 35.2%, without correlation with gender or laterality of the studied bone. These data may be supported in the literature by Leonetti et al., 40%^15^; Moreano et al., 15%^16^; Dew et al., 33%^17^; and Aslam et al., 35%^18^. The high incidence of CC dehiscence warns us about how much attention we should give during dissection in regions close to it. Preoperative complementary exams, such as computed tomography, still require further sensitivity to detect this specific type of affection. Atilla et al., studying 700 computed tomography images, detected an incidence of only 1.4% of dehiscence at axial and coronal sections ^19^, a number much smaller than what we detected in our microscopic study. In addition to the risk of intraoperative damage to ICA, CC dehiscence may also present other significant otological signs and symptoms, such as a pulsatile mass that may be taken as paragangliomas ^20^, pulsatile otorrhea, ICA aneurysm and pseudoaneurysm ^16^, hearing loss with ossicle affection or auditory tube dysfunction^21^, in addition to spontaneous otorrhagia.

The understanding of the complex anatomy of the temporal bone, involving different vital and significant anatomical structures, is still a major challenge to otological surgeons. Our study of microanatomy of the temporal bone evidencing a small distance between cochlear turns and CC and high incidence of dehiscence close to TC remind us of this fact.

## CONCLUSIONS

From the microscopic analysis of the distances between the carotid canal and cochlear turns and the occurrence of dehiscence close to the tympanic cavity in 122 human temporal bones sectioned in the horizontal plan, we concluded that:

1. There was no statistically significant difference in gender and laterality in the studied bones.
2. The smallest distance between the cochlea and the carotid canal is located most of the times in the basal turn, seldom in the medium turn and rarely in the apical turn.
3. We observed incidence of 35% of carotid canal dehiscence in the tympanic cavity.
